# Primary biliary cholangitis. Treatment options in 2025. A narrative review

**DOI:** 10.3389/fimmu.2025.1698833

**Published:** 2025-11-03

**Authors:** Maria Angelara, Klairi Papachristou, Margarita Papatheodoridi, Narjes Nasiri-Ansari, Dimitrios S. Karagiannakis, Theodoros Androutsakos

**Affiliations:** ^1^ First Department of Internal Medicine, Propaedeutic Clinic, “Laiko” Hospital, National and Kapodistrian University of Athens, Athens, Greece; ^2^ Department of Pathophysiology, Medical School, National and Kapodistrian University of Athens, Athens, Greece; ^3^ Academic Department of Gastroenterology, Medical School of National and Kapodistrian University of Athens, Athens, Greece; ^4^ Martin Luther University of Halle-Wittenberg, Halle/Saale, Germany; ^5^ Fourth Department of Internal Medicine, “Attikon” University Hospital, School of Medicine, National and Kapodistrian University of Athens, Athens, Greece

**Keywords:** autoimmune liver disease, primary biliary cholangitis, elafibranor, FXR agonists, liver, obeticholic acid, peroxisome proliferator–activated receptors, seladelpar

## Abstract

Primary biliary cholangitis (PBC) is a chronic, cholestatic disease with a female predominance and a long disease duration. The pathogenesis of PBC is still unclear; however, genetic, epigenetic, and environmental factors, alongside immune dysregulation, seem to lead to a dysfunction of the biliary ‘bicarbonate umbrella’ and increased biliary epithelial cells apoptosis. Ursodeoxycholic acid (UDCA) has been the treatment of choice for PBC since its approval back in 1994; however, a percentage varying from 15-40% of all patients fail to achieve biochemical response or alkaline phosphatase normalization. Obeticholic acid, though promising at first, failed to show benefit after long-term use and was retracted from the market. Two peroxisome proliferator–activated receptor agonists (PPARs) have recently been approved for use in patients with PBC, showing biochemical response in non-responders and improvement of pruritus. However, a substantial percentage of patients fail to achieve serum alkaline phosphatase and bilirubin normalization; as a result, many drugs with different mechanisms of action are in phase 2 or 3 trials. The aim of this review is to present available data regarding PBC treatment and explain the pathogenetic pathway each one targets.

## 1 Introduction

Primary biliary cholangitis (PBC), formerly called primary biliary cirrhosis, is a chronic cholestatic disease of autoimmune origin, presenting as destructive lymphocytic cholangitis, affecting mainly the biliary epithelial cells (BEC) of the small-bile ducts of the liver (1, 2). The disease shows a female predominance, with a female-to-male ratio of almost 10:1, and a median age of presentation of 40 years (3, 4). Environmental, genetic, and epigenetic factors have been implicated in the disease, although the exact pathogenic mechanism remains elusive (5–8). PBC usually presents with long-standing biochemical markers of cholestasis, including elevation of serum alkaline phosphatase (ALP), gamma glutamyl transferase (gGT), and bilirubin, frequently accompanied by an elevation in immunoglobulin-M concentration, while systemic manifestations like pruritus and/or fatigue and extrahepatic autoimmune manifestations like sicca syndrome, thyroid dysfunction or systemic sclerosis are not uncommon (9–11).

Antimitochondrial antibodies (AMA) targeting the E2 subunit of the pyruvate dehydrogenase complex (PDC-E2) of the inner mitochondrial membrane are highly specific for PBC and are present in more than 90% of patients, while certain antinuclear antibodies, namely antiglycoprotein 210 (anti-gp 210) and anti-sp100, occur in up to 50% of AMA negative PBC patients, highlighting the complexity of PBC pathogenesis (12–14). As a result of that, both the American and the European association of the Study of the Liver (AASLD, EASL) suggest that in patients with AMA, gp-210 and/or anti-sp100 seropositivity, the diagnosis of PBC can be safely set with no liver biopsy (15, 16).

PBC treatment has long been a clinical problem with less than 2/3 of all patients responding to first-line treatment, namely ursodeoxycholic acid (17, 18). Long-term response to treatment and monitoring progression to fibrosis/cirrhosis are central in PBC management and key point in deciding step up of treatment options. Several criteria have been developed and validated to define inadequate biochemical response after 12 months of UDCA therapy, including the Barcelona, Toronto, Paris I and Paris II, Rotterdam and POISE criteria, which mainly incorporate reductions in alkaline phosphatase (ALP) and bilirubin levels. In addition to these binary response classifications, two continuous prognostic scoring systems—the UK-PBC Risk Score and the GLOBE score—have been established and are now widely used to stratify risk and assess the efficacy of both first-line (UDCA) and second-line therapies during long-term follow-up. Interestingly enough, according to 2 metanalyses, comprising a large number of patients, UK-PBC and GLOBE scores have shown better prediction of long term cirrhosis-related complications, with similar prognostic performance (19, 20).

Recent evidence further suggests that persistent elevation of multiple liver biochemical markers (AST, ALT, ALP and bilirubin) and non-invasive fibrosis indices such as APRI and FIB-4 are associated with poorer outcomes, supporting the concept of a “deep biochemical response,” defined by complete normalization of ALP and bilirubin, as an emerging therapeutic target (21).

Lastly, transient elastography has become an important tool for disease monitoring, as both baseline liver stiffness and its progression over time correlate strongly with the risk of clinical complications, including hepatic decompensation and cirrhosis (22).

**Figure 1:**
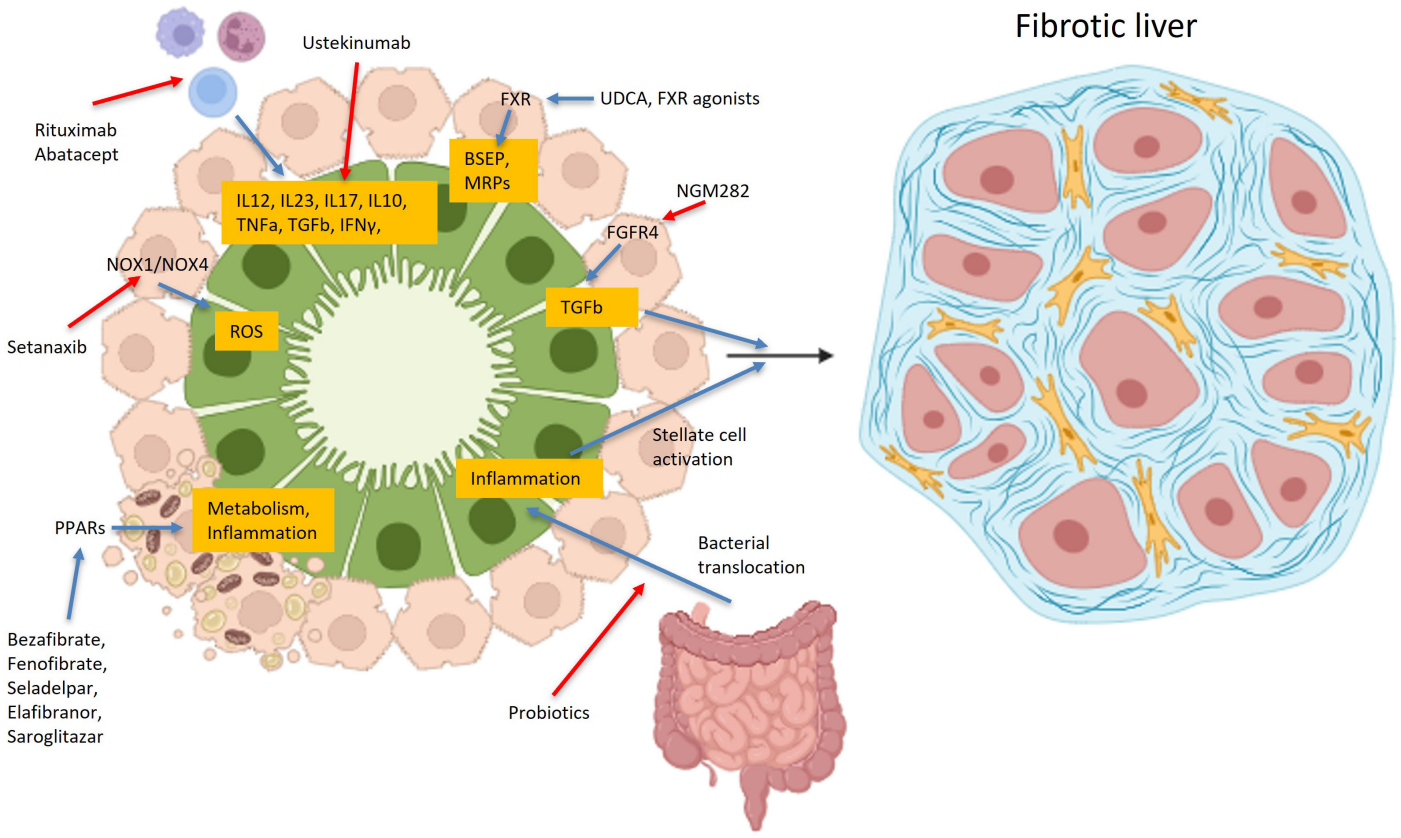
Main pathophysiological targets of various drugs used in PBC. Drugs used or in trials for primary biliary cholangitis, target a variety of different receptors, like PPAR, FXR, FGFR4, BSEP, MRP or NOX1/NOX4, bacterial microbiota, or even lymphocytes, aiming in increasing or decreasing many cytokines, like IL12, IL23, IL17, IL10 etc. Created in https://BioRender.com.

In recent years, several different drugs, targeting various disease-modifying pathways, have been used in patients with PBC with promising results. Among them, two peroxisome proliferator–activated receptor agonists (PPARs), namely Seladelpar and Elafibranor, have gained approval for use, showing biochemical response, namely alkaline phosphatase (ALP) normalization, in non-responders and improvement of pruritus. However, a substantial percentage of patients still fail to reach biochemical remission; as a result, many drugs with different mechanisms of action are in phase 2 or 3 trials (**Figure 1**).

In this review, the authors present all available treatment options for the disease, highlighting targeted pathways and clinical results of each one.

## 2 Disease mechanism

**Figure 2:**
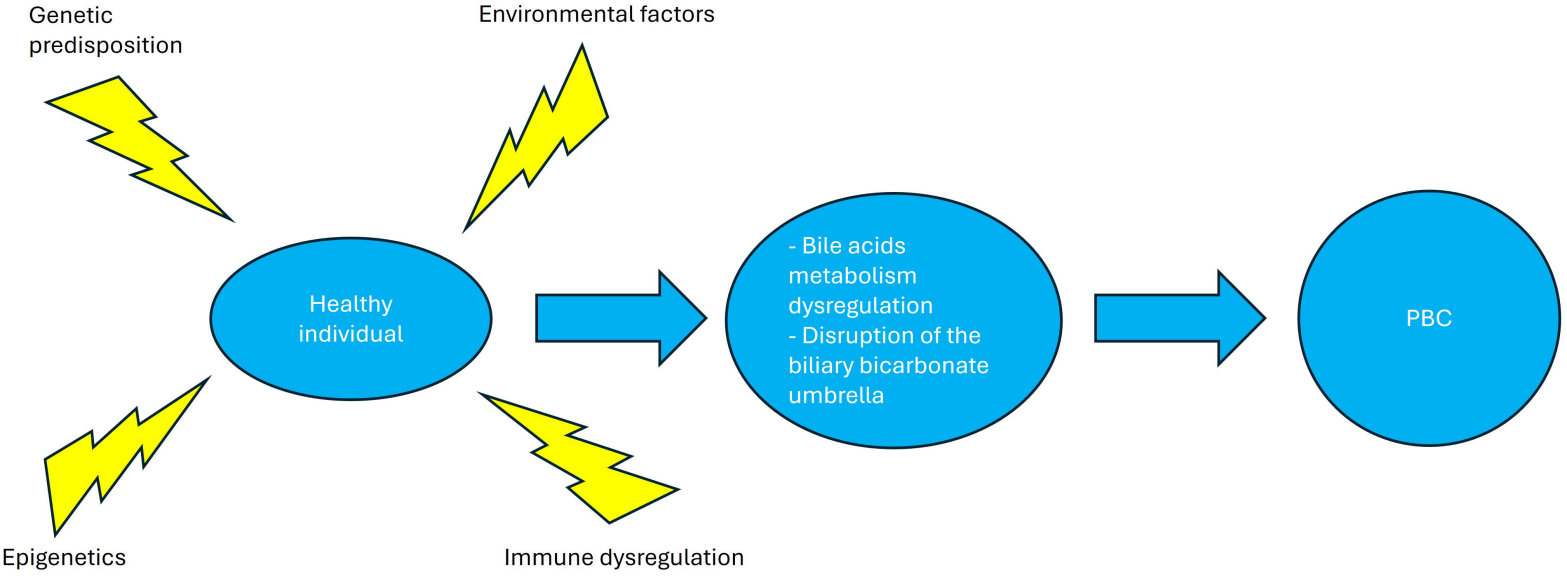
Primary biliary cholangitis pathogenesis. Environmental factors, epigenetic changes and immune dysregulation in a genetically predisposed patient lead to bile acid dysregulation and disruption of the biliary bicarbonate umbrella, leading to the clinical phenotype of primary biliary cholangitis. Created in http://BioRender.com.

The pathogenesis of PBC is largely unclear; however, evidence suggests that susceptibility to the disease results from the interplay between environmental triggers and genetic predisposition (23)with epigenetic mechanisms, immune dysregulation of both innate and adaptive immunity (**Figure 2**), leading to dysregulation of bile acids synthesis and/or metabolism of and finally disruption of the biliary bicarbonate (HCO_3_^-^) umbrella, which is critical for protecting the bile ducts from harmful bile acids through the secretion of HCO_3_^-^ into the ductular lumen. A variety of receptors regulating bile duct metabolism, such as PPAR and farnesoid-X receptors (FXR), appear to play a crucial role in disease pathogenesis, making them attractive targets for disease-modifying drugs (24).

## 3 Available treatment options

### 3.1 Current treatment regimens

#### 3.1.1 Ursodeoxycholic acid

UDCA has been the cornerstone of PBC treatment since its approval in 1994. It remains the first-line therapy and the standard of care for all patients with PBC, irrespective of liver fibrosis stage (25). UDCA is well-documented to improve liver function, slow disease progression, and enhance transplant-free survival (26, 27). Despite its widespread clinical use, research into its mechanisms of action and therapeutic efficacy continues.

**Figure 3:**
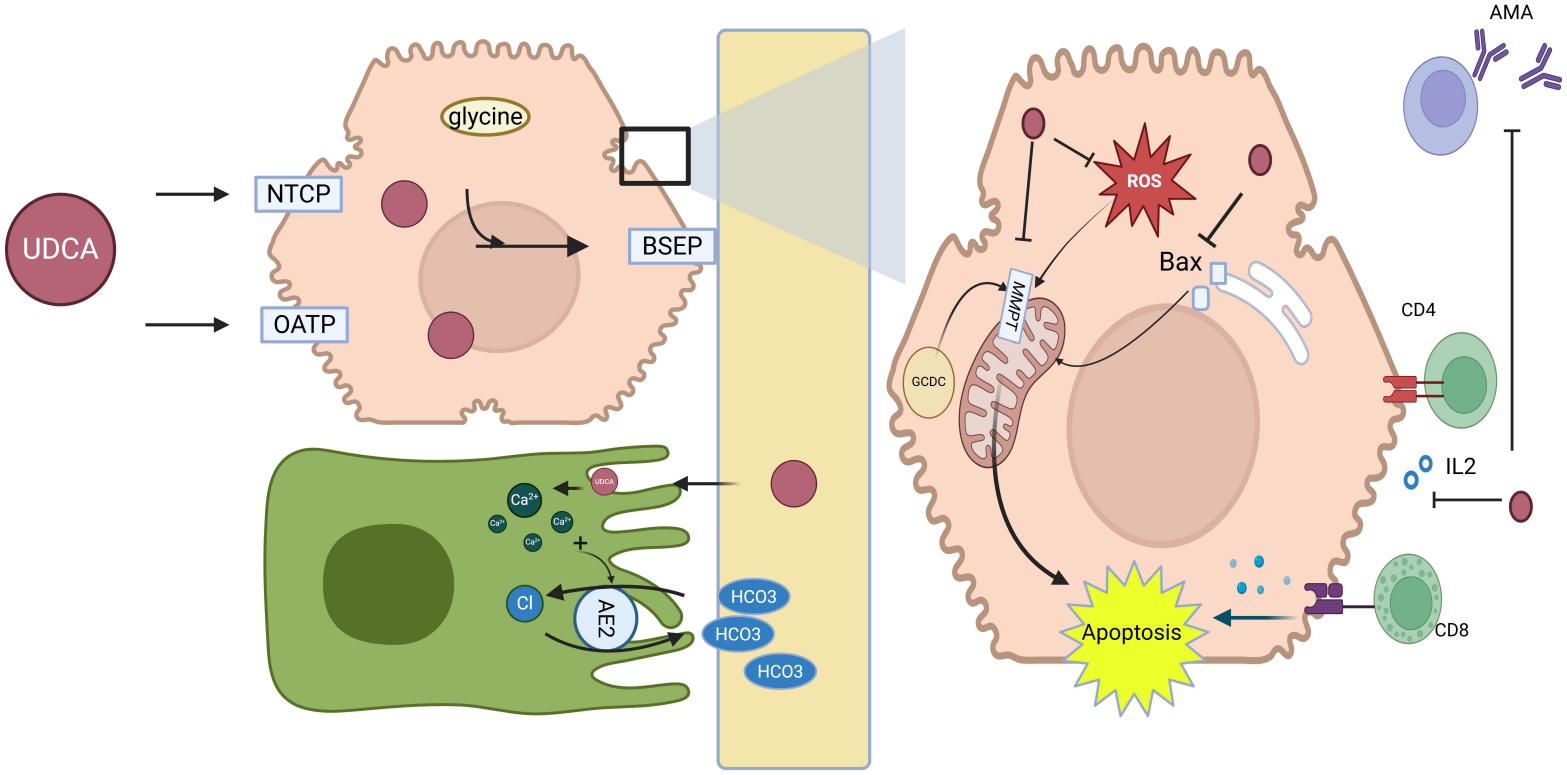
Mechanisms of action of UDCA in primary biliary cholangitis. Key molecular targets and pathways affected by UDCA, including its interaction with bile acid transporters (e.g., BSEP, MRP) and its roles in reducing bile acid toxicity, modulating immune responses, and improving liver function. UDCA is taken up by hepatocytes via NTCP and OATPs, conjugated with glycine or taurine, and secreted into bile through BSEP, promoting bile flow and displacing toxic hydrophobic bile acids. It enhances choleresis and bicarbonate secretion, stabilizes cholangiocyte and hepatocyte membranes, protects mitochondria, reduces ROS, inhibits apoptosis, and modulates immune responses by downregulating MHC expression and decreasing anti-PDH/AMA antibodies, collectively improving bile composition, alleviating cholestasis, and protecting hepatocytes in PBC. *Abbreviations*: NTCP, sodium-taurocholate co-transporting polypeptide; OATPs, organic anion-transporting polypeptides; UDCA, ursodeoxycholic acid; BSEP, bile salt export pump; AE2, anion exchanger 2; MPT, mitochondrial permeability transition; ROS, reactive oxygen species; Bax, Bcl-2-associated X protein; CD4+ T cells, cluster of differentiation 4 (helper T lymphocytes); CD8+ T cells, cluster of differentiation 8 (cytotoxic T lymphocytes); AMA, anti-mitochondrial antibodies. Created in https://BioRender.com.

UDCA is a hydrophilic bile acid naturally found in small quantities (approximately 3%) within human bile. Structurally, it is a dihydroxy bile acid, which allows it to replace the more hydrophobic and toxic bile acids that accumulate in PBC. Upon administration, UDCA enters the portal circulation and is taken up by hepatocytes through specific bile acid transporters (**Figure 3**) such as sodium-taurocholate co-transporting polypeptide (NTCP) and organic anion-transporting polypeptides (OATPs). Once inside hepatocytes, UDCA is conjugated primarily with glycine or taurine and transported into bile ducts via the bile salt export pump (BSEP), promoting bile secretion and preventing the accumulation of toxic bile acids (28).

The choleretic effect of UDCA is central to its therapeutic action in PBC. UDCA promotes bile secretion, alleviates cholestasis, and improves liver function. This is achieved through mechanisms such as increasing intracellular calcium levels, which stimulate canalicular transport and vesicular exocytosis (29, 30). Furthermore, UDCA displaces hydrophobic bile acids at both the ileal absorption site and within hepatocytes, mitigating intracellular toxicity and protecting hepatocellular and mitochondrial integrity (31).

UDCA induces bicarbonate-rich hypercholeresis by stabilizing cholangiocyte membranes and facilitating bicarbonate transport. Protonation of UDCA in bile ducts contributes to bicarbonate generation, which enhances bile flow and maintains pH balance. Additionally, the upregulation of AE2 on biliary epithelial cells further supports bicarbonate secretion, contributing to UDCA’s beneficial effects on bile composition (32). In addition to its choleretic properties, UDCA has cytoprotective, anti-inflammatory, and immunomodulatory effects, making it a multifaceted treatment option.

Furthermore, UDCA has been shown to reduce the hepatocellular and biliary expression of major histocompatibility complex (MHC) class I and II proteins, which are involved in immune-mediated liver damage, suggesting that UDCA may mitigate T-cell-mediated hepatocellular injury in PBC (33). Moreover, UDCA treatment decreases the serum levels of antibodies against pyruvate dehydrogenase (PDH) and AMA, further supporting its immunomodulatory role (34, 35).

In addition to its effects on bile secretion and immune modulation, UDCA provides direct cytoprotection to hepatocytes and biliary epithelial cells through several mechanisms. Firstly, UDCA acts by stabilizing cell membranes and reducing the toxic effects of hydrophobic bile acids on cholesterol-rich membranes. Moreover, UDCA inhibits mitochondrial membrane permeability transition (MMPT), thereby preventing apoptosis and necrosis induced by toxic bile salts such as glycochenodeoxycholic acid (GCDC). UDCA also inhibits the translocation of pro-apoptotic proteins, such as Bax, from the cytosol to mitochondria, while reducing reactive oxygen species (ROS) production and preserving mitochondrial membrane potential, further protecting hepatocytes from oxidative stress (28).

Despite its efficacy, UDCA’s impact on disease progression depends on the stage of fibrosis at the time of diagnosis and initiation of treatment. Overall, up to 40% of all patients fail to achieve adequate biochemical response, while patients diagnosed and treated at advanced fibrosis stages, even with a complete biochemical response, have a reduced transplant-free survival compared to those diagnosed at earlier stages (25, 36).

#### 3.1.2 PPAR agonists

Due to the beneficial effects of PPAR agonism in PBC natural course, as highlighted by the successful use of fibrates, several novel PPAR therapies are under investigation (**Table 1**). Several meta-analyses consistently demonstrate that PPAR agonists exert significant biochemical efficacy in UDCA-inadequate PBC, confirming their pharmacologic activity as effective disease-modifying agents (37, 38).

**Table 1:** Drugs targeting PPARs.

Drug	Key molecular targets	Role in PBC treatment
Elafibranor	PPARα/PPARδ	2^nd^ line treatment
Seladelpar	PPARδ	2^nd^ line treatment
Fenofibrate	PPARα	2^nd^ line treatment
Bezafibrate	PPARα, PPARδ, PPARγ (pan-PPAR agonist)	2^nd^ line treatment
Saroglitazar	PPARα/PPARγ	In clinical trials

PPAR, Peroxisome proliferator-activated receptor.

However, most studies have not proven yet meaningful changes in liver stiffness or ELF scores, highlighting the need for long-term data on clinical outcomes and safety so as to establish their overall therapeutic impact (39).

**Figure 4:**
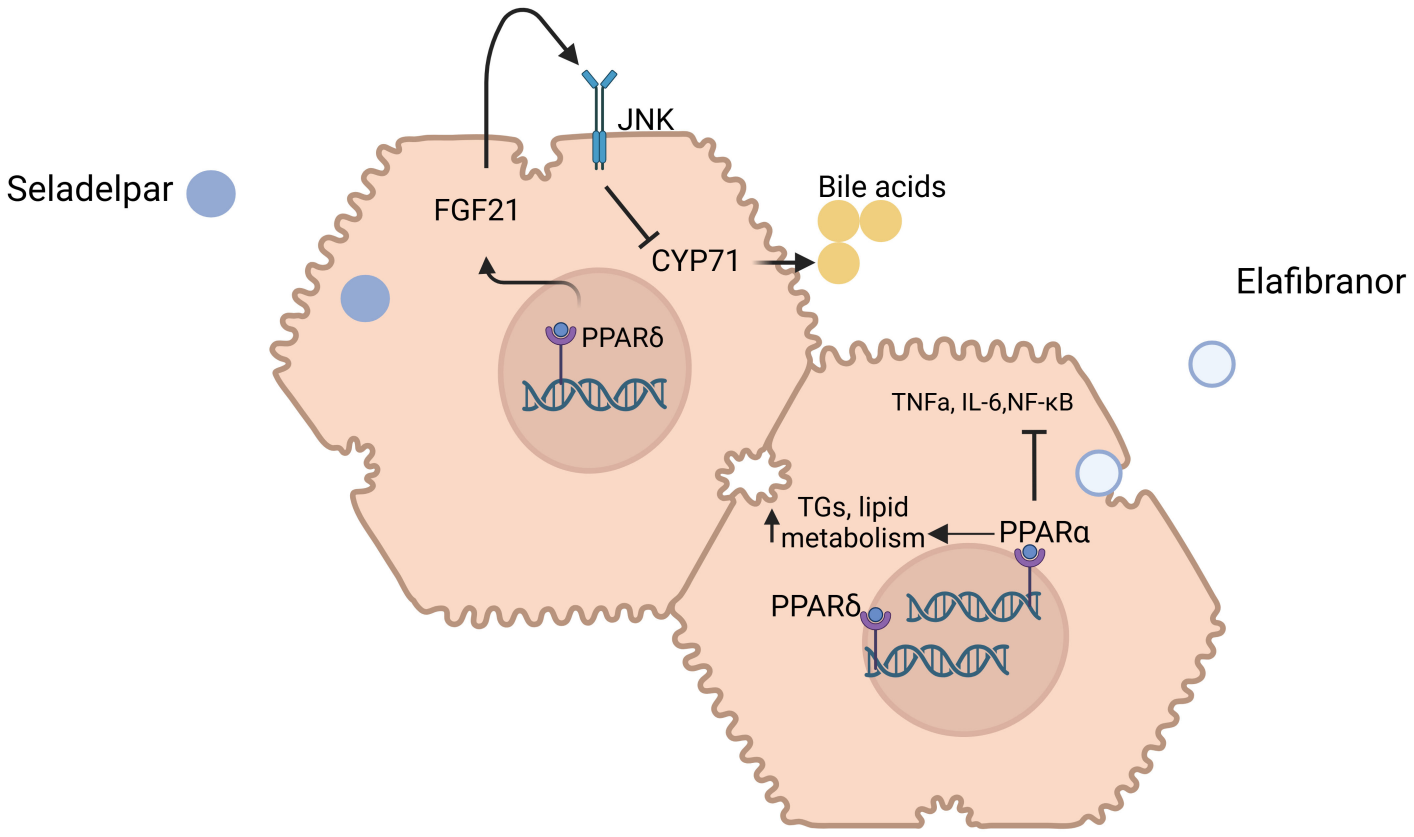
Mechanisms of action of Seladelpar and Elafibranor in primary biliary cholangitis. Molecular targets and pathways modulated by Seladelpar and Elafibranor. Seladelpar activates PPAR-δ, leading to increased levels of FGF21, which in turn downregulates CYP7A1, a key enzyme involved in bile acid synthesis. Elafibranor modulates both PPAR-α and PPAR-γ receptors to regulate lipid metabolism, inflammation, and improve liver function in PBC patients. Abbreviations: PPAR: Peroxisome Proliferator-Activated Receptor, FGF21: Fibroblast Growth Factor 21, CYP7A1: Cholesterol 7-alpha-hydroxylase, TNF-α: Tumor Necrosis Factor Alpha, IL-6: Interleukin-6, NF-κB: Nuclear Factor Kappa-Light-Chain-Enhancer of Activated B Cells. Created in http://BioRender.com.

Recently accelerated approval was given to two PPARs, namely Seladelpar and Elafibranor, for use in non-responders to UDCA as second line therapy (**Figure 4**). Both drugs have shown promising results in PBC treatment, with Elafibranor showing better results in achieving fast biochemical response and Seladelpar leading to lower incidence of pruritus according to recent meta-analyses, even though study populations were not identical (40).

##### 3.1.2.1 Seladelpar

Seladelpar, a PPAR-δ agonist, has emerged as a promising therapeutic option for the treatment of primary biliary cholangitis PBC. Unlike other PPAR isotypes, PPAR-δ is widely expressed in hepatocytes, cholangiocytes, Kupffer cells, and stellate cells, which are key players in PBC pathogenesis. Through its activation, seladelpar triggers the induction of fibroblast growth factor 21 (FGF21) from hepatocytes, activates c-Jun N-terminal kinase (JNK) signaling pathway, and subsequently inhibits cholesterol 7α-hydroxylase (CYP7A1), the rate-limiting enzyme for bile acid synthesis and accumulation; an effect occurring independently of the FXR pathway. Furthermore, seladelpar seems to improve bile acid homeostasis and exhibit potential antifibrotic effects.

In phase II, randomized, open-label 52-week study, seladelpar was evaluated at doses of 2, 5, and 10 mg for its efficacy and safety in patients with PBC. The study revealed substantial reductions in key liver function markers, such as ALP, total bilirubin, and ALT. At Week 52, the rates of composite response (defined as ALP <1.67xULN, a decrease in ALP of ≥15%, and normal total bilirubin) were 64%, 53%, and 67% for the 2 mg, 5 mg, and 10 mg cohorts, respectively. Additionally, the rates of ALP normalization were 9%, 13%, and 33% for the same cohorts. Moreover, in a predefined subgroup analysis, reductions in ALP, total bilirubin, and ALT were comparable between patients with and without cirrhosis at weeks 12 and 52. In the 10mg cohort, ALP levels decreased by 48.5% in patients with cirrhosis and by 43.2% in those without, highlighting the drug’s effectiveness irrespective of cirrhosis status (41).

The most common (≥10%) adverse event was pruritus (24.4%); however, of the 29 patients reporting it, 18 were already suffering from it at trial inclusion. A significant limitation of this study is the lack of standardized criteria for dosage titration after week 12. This inconsistency introduces variability in the treatment regimen, making it challenging to directly compare outcomes across cohorts beyond this time point.

In addition to these biochemical improvements, a separate phase II open-label trial demonstrated seladelpar’s broader benefits, including significant reductions in pruritus, sleep disturbances, and fatigue. Over the course of one year of treatment, seladelpar decreased serum bile acid and bilirubin levels, while improving patients’ overall quality of life (42).

The efficacy of seladelpar was highlighted in the RESPONSE Phase 3 trial, a 12-month, double-blind, placebo-controlled study involving 193 PBC patients, received either seladelpar or placebo, with background standard of care therapy of UDCA. In this trial, 61.7% of patients receiving seladelpar achieved a biochemical response compared to 20.0% of those on placebo. Additionally, 25% of seladelpar-treated patients achieved ALP normalization, compared to none in the placebo group. Major adverse events were infrequent in both groups. Notably, seladelpar demonstrated a significant reduction in pruritus, especially among patients with moderate-to-severe pruritus at baseline, a finding that contrasts with the pruritus often seen with OCA, the only FDA-approved second-line therapy for PBC. On the other hand, no meaningful changes in liver stiffness or ELF scores were found (43).

Safety data from the trial indicated that seladelpar was generally well-tolerated, with adverse events such as abdominal pain, headache, and nausea being more common in the seladelpar group compared to the placebo group, although the incidence of serious adverse events was similar between the two groups. Notably, patients with advanced PBC, including those with hepatic decompensation, were excluded from the study (43).

Based on the above trial, the U.S. Food and Drug Administration (FDA) granted seladelpar accelerated approval for PBC on August 14, 2024, recognizing its effectiveness in reducing bile acid synthesis and improving biochemical markers.

On November 2024, the interim results from the ongoing, open-label Phase 3 ASSURE study were published. Lawitz et al. reported a biochemical response in 81% of patients at month 30 as well as a significant reduction in pruritus at 6 months. Furthermore, the study highlights the long-term safety profile of seladelpar, with the most common adverse events being COVID-19, pruritus and nausea (44).

##### 3.1.2.2 Elafibranor

Elafibranor is a dual agonist of PPARα and PPARβ/δ, offering a range of metabolic benefits. Through PPARα activation, it reduces triglyceride levels and promotes lipid metabolism, while PPARβ/δ activation enhances fatty acid transport and oxidation, improves glucose homeostasis, and further increases high-density lipoprotein (HDL) cholesterol levels. These combined actions position elafibranor as a potential therapeutic agent for metabolic and lipid-related disorders. Moreover, elafibranor, like seladelpar, reduces bile acid synthesis through FGF21-mediated suppression of CYP7A1 (45).

The Phase 3 ELATIVE trial (NCT06016842), a double-blind, randomized, placebo-controlled study, evaluated data on long-term elafibranor’s safety and efficacy in PBC patients who had an inadequate response to UDCA (46). The trial involved patients receiving elafibranor or placebo while continuing UDCA therapy. At 52 weeks, 51% of patients in the elafibranor group achieved a significant biochemical response, compared to just 4% in the placebo group—demonstrating an impressive difference of 47% (95% CI, 32 to 57; P<0.001). This response was characterized by rapid and sustained ALP reduction.

One of the most promising findings from the ELATIVE trial was the early improvement in ALP levels, seen as soon as four weeks after drug initiation. This rapid reduction persisted throughout the 52-week treatment period. Importantly, 15% of patients treated with elafibranor achieved full normalization of ALP, compared to none in the placebo group.

Despite the potential improvement in pruritus in patients treated with elafibranor, as assessed by tools like the PBC-40 questionnaire and the 5-D itch scale, the primary measurement of pruritus (WI-NRS) did not show significant differences between the elafibranor and placebo groups. Additionally, in patients with moderate-to-severe baseline fatigue, elafibranor produced a statistically significant improvement compared to placebo, with 66.7% achieving a clinically meaningful reduction versus 31.3% in the placebo group.

Elafibranor was generally well tolerated. However, four patients discontinued treatment due to elevated creatine phosphokinase levels, underscoring the importance of regular monitoring.

As a result, on June 10, 2024, the U.S. Food and Drug Administration (FDA) granted elafibranor accelerated approval for PBC, marking a pivotal advancement in the management of this chronic autoimmune liver disease, while EMA conditional approval was obtained on September 19, 2024.

Furthermore, in the ELATIVE open-label extension trial, the results by week 156 demonstrated sustained efficacy and tolerability of elafibranor. Biomarkers of cholestasis, including ALP and total bilirubin, remained improved, while fibrosis surrogates, such as median liver stiffness measurement (LSM) and Enhanced Liver Fibrosis (ELF) score, remained stable over the same period. Among patients with moderate-to-severe baseline fatigue, 56% experienced a clinically meaningful improvement, highlighting the sustained benefit on this symptom (47).

#### 3.1.3 Obeticholic acid

The approval of OCA as a second-line treatment in 2016 represented a breakthrough, since it offered a vital alternative for patients with limited treatment options, filling a critical gap in PBC management.

OCA is a semisynthetic hydrophobic bile acid analog that selectively binds to a Farnesoid X receptor (FXR), expressed mostly in enterocytes and hepatocytes. This potent agonist of FXR triggers the release of FGF19 from ileal enterocytes, which then acts on hepatocytes by binding to FGFR4 and suppressing CYP7A1 expression, thus leading to a reduction in bile acid synthesis and secretion (48, 49). Studies have shown additional effects of OCA treatment by suppressing bile acid synthesis genes (CYP7A1, CYP27A1) and increasing basolateral efflux transporter genes (ABCB4, ABCB11, OSTA, OSTB) (50). FXR activation exerts anti-inflammatory and antifibrotic actions by modifying the activities of PPARγ and SHP, with a corresponding decrease in the profibrotic activities of a1 collagen, TGF-b1 and NLRP3 inflammasome activation (51–54).

In 2016, OCA was conditionally approved for use in PBC patients based on a phase 3 double blind, placebo-controlled study (POISE) where 216 patients with inadequate response or intolerance to UDCA were randomized to receive at least one dose of OCA or placebo for 12 months. The response to OCA was substantial, with 46% of patients in the titration group (5–10 mg) and 47% in the 10 mg group, achieving an alkaline phosphatase level less than 1.67 times the upper limit of normal, with a reduction of at least 15% from baseline, along with a normal total bilirubin level, compared to only 10% in the placebo group (P<0.001 for both) (55). OCA was generally well tolerated; however, pruritus, reported in up to 77% of patients in a dose-dependent manner, was the most common adverse effect, followed by fatigue, which occurred in approximately 33% of patients. Similar results were demonstrated in a recent meta-analysis combining data from observational studies and RCTs (56).

However, the results of a confirmatory, phase 3b/4, randomized controlled trial of OCA (COBALT) assessing the hard endpoints of time to death, liver transplant, model for end-stage liver disease score ≥15, uncontrolled ascites, or hospitalization for hepatic decompensation were rather disappointing. More specifically, patients assigned to receive OCA (5–10 mg) were compared either with a placebo in a randomized controlled trial (RCT) or with an external control (EC). No differences were found between the OCA and placebo group, leading to a premature study ending due to functional unblinding and treatment crossover in December 2021 (57).

Based on the above results, European Medicines Agency (EMA) concluded that benefits did not outweigh risks in the therapy with OCA and proposed the drug approval to be withdrawn in the EU. Moreover, the FDA released a warning that the use of OCA in decompensated cirrhosis was associated with clinical worsening and subsequent absolute contraindication of its use in these patients, without revoking the drug license (58). However, in February 2025, the COBALT trial published results using different statistical analyses including external controls and adjusted models, showing a significant reduction in occurrence of hard clinical outcomes (57). These findings highlight the complexity and uncertainty surrounding OCA’s role in the management of PBC.

As a result, the initial excitement about OCA was tempered, once again highlighting the need for new, effective treatments for patients with PBC.

#### 3.1.4 Fibrates

Fibrates, as PPAR agonists, have become popular adjunctive therapies for patients not fully responsive to UDCA, contributing both to disease progression and the accompanying pruritus and are now recommended as second-line, off-label, treatment for PBC (15). Among these, bezafibrate and fenofibrate are the most widely used.

##### 3.1.4.1 Bezafibrate

Bezafibrate acts as an agonist for PPARα, β, and δ, as well as the PXR, showing effects in both bile acid metabolism and inflammatory pathways. Bezafibrate reduces hepatic bile acid concentration by down-regulating key bile acid transporters (NTCP, CYP7A1, and CYP27A1) and upregulating basolateral bile acid efflux transporters such as MRP4. These changes result in decreased bile acid accumulation in hepatocytes, reducing bile acid-mediated cytotoxicity. Bezafibrate, through PXR-activation upregulates MDR1, MRP2, and CYP3A4, which facilitate the elimination of toxic bile acids, organic anions, and xenobiotics from hepatocytes to bile canaliculi. These anticholestatic effects are further complemented by the anti-inflammatory properties of the drug, which inhibit NF-κB and proinflammatory cytokines such as TNF-α (59).

Bezafibrate has been shown to significantly improve biochemical markers of liver function in patients with PBC, particularly those who have an inadequate response to UDCA alone. In a 2018 randomized controlled trial conducted in France, patients with incomplete response to UDCA were assigned to receive either bezafibrate (400 mg/day) or placebo for 24 months. This trial demonstrated that 31% of patients in the bezafibrate group achieved a complete biochemical response—defined as normalization of key liver enzymes—compared to 0% in the placebo group (P<0.001). This improvement was sustained throughout the 24-month trial period and was accompanied by reductions in liver stiffness and enhanced liver fibrosis (ELF) score. However, in a subgroup of patients with available histologic data (26 in the bezafibrate group and 25 in the placebo group), changes in fibrosis stage and activity grade were not statistically significant between the groups. Also, features of portal hypertension developed in similar proportion of patients in both groups, suggesting that bezafibrate may not be as effective in advanced stages. The trial’s small size and duration were insufficient to evaluate bezafibrate’s impact on major outcomes (60).

However, further evidence from a large retrospective study of 3,908 patients in Japan supported the use of bezafibrate as a second-line therapy in PBC. This study showed that combining bezafibrate with UDCA significantly reduced all-cause mortality, liver-related death, and the need for liver transplantation compared to UDCA monotherapy. The adjusted hazard ratio (HR) for all-cause mortality or liver transplant was 0.325 (P<0.001), and the HR for liver-related death or transplant was 0.274 (P<0.001) (61).

Despite its efficacy, bezafibrate is associated with potential adverse effects, particularly in patients with advanced liver disease or renal impairment. Common side effects include increased serum creatinine levels, rhabdomyolysis, and hepatotoxicity. Long-term combination therapy with UDCA and bezafibrate has been associated with a significant increase in serum creatinine levels, necessitating careful monitoring of renal function during treatment. Bezafibrate is also contraindicated in patients with decompensated cirrhosis (Child-Pugh class B or C), as the American Association for the Study of Liver Diseases (AASLD) guidelines advise against fibrate use in these populations (58).

Overall, bezafibrate represents a promising adjunct therapy for PBC patients who have an incomplete response to UDCA. Its ability to improve liver biochemistry and reduce mortality risk has been well demonstrated; however, safety concerns, particularly regarding renal function and patients with cirrhosis, must be carefully managed.

##### 3.1.4.2 Fenofibrate

Fenofibrate is a PPAR-α agonist that plays a pivotal role in the regulation of lipid metabolism, fatty acid oxidation, and bile acid homeostasis. Activation of PPAR-α by fenofibrate enhances the clearance of toxic bile acids, thereby reducing their hepatic accumulation and mitigating cholestasis. Additionally, fenofibrate exerts anti-inflammatory effects by downregulating the production of pro-inflammatory cytokines such as TNF-α and interleukins, which contribute to liver injury in PBC. Its antioxidant properties further protect hepatocytes by reducing oxidative stress, thus providing additional hepatoprotective benefits.

Several studies have investigated the effectiveness of fenofibrate in treating PBC, particularly in patients who show an incomplete response to UDCA.

In a randomized clinical trial by Liu et al., fenofibrate was evaluated in treatment-naive PBC patients. The study compared the efficacy of UDCA combined with fenofibrate versus UDCA alone. At 12 months, 81.4% of patients in the UDCA-fenofibrate group achieved a biochemical response based on the Barcelona criterion, compared to 64.3% in the UDCA-only group (p = 0.048). Although the primary outcome showed a statistically significant improvement, the study did not find any significant differences in non-invasive measures of liver fibrosis between the two groups during the same period (62).

Fenofibrate is generally well-tolerated in patients with PBC, though it has been associated with some adverse effects, including gastrointestinal discomfort, muscle pain (myalgia), and elevations in serum creatinine, reflecting potential kidney stress. Monitoring renal function is crucial, especially in patients with pre-existing renal impairment or those undergoing combination therapy with statins. One rare but serious complication associated with fenofibrate is rhabdomyolysis, a risk that is particularly elevated when statins are co-administered. Additionally, fenofibrate is contraindicated in patients with decompensated cirrhosis (Child-Pugh class B or C) due to a higher risk of hepatotoxicity.

#### 3.1.5 Budesonide

Budesonide, a second-generation glucocorticoid steroid, has gained attention as a therapeutic option in the management of PBC due to its targeted anti-inflammatory and immunomodulatory properties. It primarily acts as a glucocorticoid receptor agonist, leveraging its extensive first-pass metabolism in the liver to deliver localized anti-inflammatory effects while minimizing systemic exposure. This pharmacokinetic profile makes budesonide particularly suitable for conditions such as PBC, where localized hepatic inflammation plays a central role in disease pathogenesis. Additionally, budesonide inhibits the activation and migration of inflammatory cells, including lymphocytes and macrophages, which play pivotal roles in PBC pathogenesis.

Three randomized controlled trials (RCTs) have investigated the use of budesonide in PBC. Overall, these trials have shown improvement of mainly hepatic inflammation, while the improvement in fibrosis is not consistent (63–65).

Budesonide seems to be safe at lower doses, with mild gastrointestinal symptoms and rare adrenal suppression, unlike systemic corticosteroids, probably due to its high first-pass hepatic metabolism; adverse effects seem to occur more commonly in patients with cirrhosis and in increased dosage (66).

Despite its potential benefits, the use of budesonide in PBC remains somewhat controversial and is not universally recommended in current clinical guidelines. The American Association for the Study of Liver Diseases (AASLD) and the European Association for the Study of the Liver (EASL) guidelines suggest considering budesonide in patients with inadequate response to UDCA or those intolerant to UDCA, particularly in the absence of other viable treatment options (16, 59). As a result, budesonide is mainly used in patients with overlapping autoimmune hepatitis features, in conjunction with anti-cholestatic therapy with UDCA, obeticholic acid (OCA), and/or fibrates.

### 3.2 Treatment regimens under clinical trials

#### 3.2.1 Other PPARs

##### 3.2.1.1 Saroglitazar

In line with seladelpar and elafibranor, saroglitazar, a dual peroxisome PPAR-α/γ agonist, is under trials for use in PBC (**Table 2**).

**Table 2:** Ongoing clinical trials.

NCT number	Drug	Study status	Study phase	Study type
NCT06051617	Seladelpar	Recruiting	Phase3	Interventional
NCT06798454	PVT201	Recruiting	Phase1	Interventional
NCT05896124	CS0159	Active_Not_Recruiting	Phase2	Interventional
NCT03301506	Seladelpar	Active_Not_Recruiting	Phase3	Interventional
NCT06060665	Seladelpar	Recruiting	Phase3	Interventional
NCT06755151	Fenofibrate	Recruiting	Phase3	Interventional
NCT06755541	Fenofibrate/UDCA	Recruiting	Phase3	Interventional
NCT06365424	Fenofibrate	Recruiting	Phase2|Phase3	Interventional
NCT06016842	Elafibranor	Recruiting	Phase3	Interventional
NCT06730061	Elafibranor	Recruiting	Phase3	Interventional
NCT06427395	Saroglitazar	Recruiting	Phase3	Interventional
NCT05050136	Volixibat	Recruiting	Phase2	Interventional
NCT04526665	Elafibranor	Active_Not_Recruiting	Phase3	Interventional
NCT04594694	OCA/Bezafibrate	Active_Not_Recruiting	Phase2	Interventional
NCT05133336	Saroglitazar	Active_Not_Recruiting	Phase2|Phase3	Interventional
NCT04514965	Bezafibrate	Recruiting		Observational
NCT06383403	Elafibranor	Recruiting	Phase3	Interventional
NCT05239468	OCA/Bezafibrate	Active_Not_Recruiting	Phase2	Interventional
NCT06247735	Pemafibrate	Recruiting	Phase2	Interventional
NCT06825559	Saroglitazar	Not_Yet_Recruiting	Phase1	Interventional
NCT06174402	Fenofibrate/UDCA	Recruiting	Phase2|Phase3	Interventional
NCT06371196	Babaodan	Not_Yet_Recruiting	Phase4	Interventional
NCT05749822	Fenofibrate	Recruiting	Phase2|Phase3	Interventional
NCT06447168	Elafibranor	Recruiting		Observational
NCT05751967	Fenofibrate/UDCA	Recruiting	Phase3	Interventional
NCT05104853	CNP-104	Active_Not_Recruiting	Phase1|Phase2	Interventional
NCT06443606	Bezafibrate	Not_Yet_Recruiting	Phase3	Interventional
NCT06888115	CS0159	Not_Yet_Recruiting	Phase1	Interventional
NCT06525311	Pemafibrate	Recruiting	Phase1	Interventional
NCT06417398	UTAA09	Not_Yet_Recruiting	Early_Phase1	Interventional

Data provided from ongoing clinical trials as of 11/04/2025. Study status and phase may be subject to change. For the most up-to-date information, please consult the clinical trial registry (ClinicalTrials.gov).

In a proof-of-concept, placebo-controlled phase 2 randomized controlled trial (RCT) by Vuppalanchi et al. in 2022, 37 patients with PBC who were already receiving UDCA were assigned to receive either 4 mg or 2 mg of saroglitazar or placebo. Results indicated that saroglitazar significantly reduced alkaline phosphatase (ALP) levels after just 4 weeks of treatment, with sustained efficacy over a 16-week period. This rapid and sustained reduction in ALP, a key biomarker for PBC, suggests that saroglitazar may be an effective treatment for patients with inadequate responses to UDCA (67).

However, the study also raised concerns about dose-dependent adverse events, particularly drug-induced liver injury (DILI), which occurred more frequently in the 4 mg saroglitazar group. Moreover, the trial did not provide conclusive evidence regarding pruritus due to the low prevalence of pruritus among participants.

To address these concerns, a Phase 3 randomized controlled trial (RCT) known as EPICS-III (NCT05133336) is currently in progress. This study compares lower doses of saroglitazar (2 mg and 1 mg) with placebo to further assess the drug’s safety and efficacy in a larger cohort of patients with PBC.

#### 3.2.2 FXR agonists

As already mentioned, FXR plays a pivotal role in primary biliary cholangitis (PBC) by regulating bile acid synthesis and transport, which are critical for maintaining liver homeostasis. Following the approval of OCA as a second-line regimen, more FXR-agonists have been tested as potential treatment regimens, yielding promising results (**Table 3**).

**Table 3:** Drugs targeting FXRs.

Drug	Key molecular targets	Role in PBC treatment
OCA	FXR	2^nd^ line treatment
Tropifexor	FXR	In clinical trials
Cilofexor	FXR	In clinical trials
EDP-305	FXR	In clinical trials

FXR, Farnesoid X receptor.

##### 3.2.2.1 Tropifexor

Tropifexor is a non-steroidal carboxylic acid investigational drug that acts as a selective FXR agonist.

In a randomized, double-blind study evaluating tropifexor, 61 patients received varying doses (30, 60, 90, or 150 μg) or placebo for 28 days (68). The results showed a significant reduction in ALP and GGT levels compared to placebo, although normalization of ALP levels was achieved in only a few patients. However, the study’s short duration of 28 days limits the strength of the conclusions that can be drawn. Additionally, the FXR-mediated induction of ALP gene transcription may complicate the interpretation of these ALP reductions.

On the other hand, tropifexor seemed to be safe, with few side effects apart from dose-dependent pruritus.

##### 3.2.2.2 Cilofexor

Cilofexor is another synthetic, non-steroidal, highly selective FXR agonist designed for the treatment of PBC.

In a randomized, double-blind trial, 71 PBC patients were given either cilofexor 30mg or 100mg or a placebo drug once daily for 12 weeks. Patients receiving cilofexor, particularly at the 100 mg dosage, exhibited significant reductions in ALP, GGT, and bile acids levels compared to placebo. Notably, 14% of patients in the 100 mg group and 9% in the 300 mg group achieved the target endpoint of ALP levels below 1.67 times the upper limit of normal. However, treatment discontinuation due to pruritus was observed in these groups, emphasizing the need for further studies (69).

##### 3.2.2.3 EDP-305

EDP-305 is a non-bile acid FXR agonist with minimal activity against TGR5. EDP-305 is currently being evaluated in a phase 2, randomized, double-blind, placebo-controlled trial. Participants will receive either EDP-305 or placebo for 12 weeks, with the primary endpoint being a 20% reduction or normalization of ALP at the end of treatment [NCT03394924].

#### 3.2.3 FXR-FGF19 pathway

##### 3.2.3.1 NGM282

FGF19 is a protein synthesized in the intestine in response to FXR activation and bile acid stimulation. It subsequently travels to the liver, where it binds to the FGFR4 receptor, inhibiting bile acid synthesis by suppressing CYP7A1 transcription (70). Notably, FGF19 has been associated with tumorigenic potential through activation of the STAT3 pathway, as demonstrated in mouse models. To mitigate this risk, an engineered analogue, FGF19(70), has been developed (71).

In a phase 2 trial assessing NGM282, an engineered analogue of FGF19(70), patients with PBC who had an inadequate response to UDCA exhibited significant reductions in alkaline phosphatase (ALP) levels after 28 days of treatment. The study reported that 50% of patients receiving NGM282 at 0.3 mg and 46% at 3 mg achieved a ≥15% reduction in ALP levels, with no exacerbation of pruritus and an acceptable safety profile. Overall, NGM282 led to significant improvements in ALP and transaminase levels; importantly, NGM282 treatment did not increase the incidence or severity of pruritus, underscoring the drug’s potential as a therapeutic option for PBC (72).

#### 3.2.4 Immunoregulatory target therapies

##### 3.2.4.1 S-adenosyl-L-methionine

S-adenosyl-L-methionine (SAMe) is an endogenous molecule with hepatoprotective properties, primarily mediated through redox regulation and methylation. In a study involving 17 female patients with primary biliary cholangitis (PBC) (mean age: 54 ± 7.9 years), administration of SAMe at a dose of 1200 mg daily for 24 weeks yielded promising results. Serum samples, collected at five different time points, demonstrated a reduction in serum anti-mitochondrial autoantibody (AMA-M2) titers and improvements in liver biochemistry in nine patients classified as SAMe responders. The protective effects of SAMe were further substantiated *in vitro*, where it safeguarded cholangiocytes from oxidative stress through the TNFα/Nrf-2/HO-1 pathway, reduced apoptosis, and enhanced protein S-glutathionylation by upregulating glutathione (GSH) synthesis enzymes. These findings suggest that SAMe may mitigate autoimmune responses in PBC via its antioxidant properties (73).

##### 3.2.4.2 Setanaxib (GKT137831)

NADPH oxidases (NOX), particularly NOX1 and NOX4, play a critical role in amplifying inflammatory and fibrotic pathways, thereby contributing to liver fibrosis through their effects on hepatocytes, fibroblasts, and endothelial cells. GKT137831, a novel small-molecule inhibitor targeting NOX1 and NOX4, has demonstrated potent anti-inflammatory and anti-fibrotic effects in preclinical studies. In animal models of acute biliary injury and steatohepatitis, GKT137831 reduced liver fibrosis, hepatocyte apoptosis, and reactive oxygen species production, highlighting its therapeutic potential in mitigating liver damage (74).

Setanaxib (GKT-831), an experimental dual inhibitor of NOX1 and NOX4, was evaluated in a Phase 2 randomized, multicenter clinical trial (NCT03226067) involving PBC patients who had been on ursodeoxycholic acid (UDCA) therapy for at least six months. Participants received setanaxib at doses of 400 mg once or twice daily, or a placebo, alongside UDCA for 24 weeks. While the primary endpoint (percentage change in gamma-glutamyl transferase [GGT] from baseline at Week 24) was not met, secondary endpoint data, including changes in alkaline phosphatase (ALP), liver stiffness, and fatigue, suggested potential anti-cholestatic and anti-fibrotic effects, warranting further investigation in subsequent trials (75). A *post hoc* analysis of the trial revealed a notable reduction in fatigue, particularly in patients with moderate to severe baseline fatigue, further supporting the potential benefits of setanaxib in this subgroup (76).

The ongoing TRANSFORM study (NCT05014672), a Phase 2b/3 trial, aims to evaluate the safety and efficacy of setanaxib at higher doses (1200 mg/day and 1600 mg/day) over 52 weeks in patients with PBC who have significant liver stiffness and an inadequate response or intolerance to UDCA.

##### 3.2.4.3 Rituximab

Given the pivotal role of immune dysregulation in PBC pathogenesis, B cell-directed therapies represent a logical treatment strategy. While preclinical studies in murine models have shown encouraging results, these findings have not yet been consistently replicated in clinical trials.

In a murine study, anti-CD20 therapy significantly reduced liver inflammation and the incidence of autoimmune cholangitis, particularly when initiated early (at 4–6 weeks of age). This early intervention led to a notable reduction in activated hepatic CD8+ T cells. Conversely, the therapeutic impact of anti-CD20 therapy was markedly reduced when administered at later stages (20–22 weeks of age), demonstrating limited efficacy on both liver and colon inflammation. These findings underscore the importance of timing in the success of B-cell-targeted interventions (77).

Clinical investigations of B-cell depletion have also been conducted in PBC. Rituximab, a monoclonal anti-CD20 antibody, was evaluated in patients refractory to ursodeoxycholic acid (UDCA) therapy. While rituximab demonstrated safety and a significant reduction in autoantibody production, its biochemical efficacy was limited. A small proportion of patients achieved normalization or substantial improvement in alkaline phosphatase (ALP) levels, suggesting that while rituximab may reduce autoimmune activity, its impact on overall liver function in PBC patients is modest (78).

##### 3.2.4.4 Ustekinumab

Whole-genome sequencing studies have identified significant associations between PBC and common genetic variants at the HLA class II, IL12A, and IL12RB2 loci. These findings underscore the relevance of the interleukin-12 (IL-12) immunoregulatory signaling axis in the pathophysiology of PBC (79).

Ustekinumab, a human IgG1 kappa monoclonal antibody, targets the shared p40 subunit of IL-12 and IL-23, inhibiting their interaction with the IL12Rβ1 receptor on cell surfaces. This mechanism was explored in an open-label trial involving 20 PBC patients with an incomplete response to ursodeoxycholic acid (UDCA). Participants received subcutaneous injections of ustekinumab (90 mg) at weeks 0 and 4, followed by injections every 8 weeks through week 20. Although the trial’s primary endpoint—a 40% reduction in ALP from baseline—was not achieved, the study provided valuable insights into the pathway’s role in PBC pathogenesis and emphasized the need for further investigation to optimize therapeutic strategies (80).

##### 3.2.4.5 Baricitinib

A whole-genome sequencing study in PBC patients identified several candidate genes, including ACT1, PIN1, DNMT1, and NTN1, suggesting their involvement in immune signaling pathways such as the IL-17, NF-κB, IL-6, JAK-STAT, IFN-γ, and TGF-β pathways. Baricitinib, a JAK1 and JAK2 inhibitor widely used for rheumatoid arthritis, specifically targets the JAK-STAT pathway, which is implicated in autoimmune processes (81).

A randomized, double-blind, placebo-controlled trial evaluated baricitinib (2 mg/day) in PBC patients with inadequate response to ursodeoxycholic acid (UDCA). However, due to extremely low enrollment (only two patients), no definitive conclusions could be drawn. Larger studies with diverse cohorts are required to ascertain the efficacy and safety of baricitinib in PBC management (82).

##### 3.2.4.6 Abatacept

Abatacept, a CTLA-4 IgG antibody used in rheumatoid and psoriatic arthritis, inhibits T-cell activation by binding to CD80 and CD86 on antigen-presenting cells (APCs). By mimicking the natural CTLA-4 mechanism, it disrupts CD28-mediated co-stimulatory signals, which are essential for naive T-cell activation. Preclinical studies in murine models showed that CTLA-4/Ig significantly reduced intrahepatic T-cell infiltrates and bile duct damage, indicating potential efficacy in PBC (83).

In an open-label trial (NCT02078882), 16 PBC patients received abatacept (125 mg subcutaneous injections weekly) for 24 weeks. Although well tolerated, the trial did not achieve the desired biochemical responses, as only one patient showed normalization or a >40% reduction in ALP levels. These findings highlight the limited impact of abatacept on clinical outcomes in PBC, warranting further exploration (84).

##### 3.2.4.7 Anti-fractalkine antibody

E6011, an antibody targeting fractalkine (CX3CL1), inhibits the CX3CL1-CX3CR1 axis, which drives lymphocyte recruitment and inflammation. In PBC, bile duct injury upregulates CX3CL1 expression in biliary epithelial cells, attracting CD4+ and CD8+ T cells and intraepithelial lymphocytes. A randomized trial in healthy Japanese men demonstrated that E6011 was safe and well tolerated, although its efficacy in PBC remains unexplored (85).

#### 3.2.5 Probiotics

Probiotics are emerging as potential therapeutic agents for PBC due to their ability to modulate gut microbiota and influence immune responses. Early studies suggest that probiotics may improve bile acid metabolism and reduce liver fibrosis in chronic liver diseases.

In a murine model of liver fibrosis, Lactobacillus rhamnosus GG (LGG) treatment reduced liver inflammation, injury, and fibrosis by modulating the gut-liver axis, particularly the FXR-FGF19 signaling pathway. LGG inhibited hepatic bile acid synthesis and enhanced bile acid excretion, highlighting its potential in targeting liver fibrosis through bile acid metabolism modulation (86).

Similarly, Lactobacillus acidophilus demonstrated efficacy in a murine model of cholestatic liver injury by attenuating liver damage, inhibiting bile acid synthesis via the CYP7A1 and FGF15 pathways, and promoting bile acid excretion. A clinical trial in humans corroborated these findings, showing that L. acidophilus improved liver function and altered bile acid profiles and gut microbiota composition. These studies suggest that L. acidophilus may be a promising therapeutic option for cholestatic liver diseases, including PBC, although further clinical trials are necessary to confirm its efficacy and safety (87).

## 4 Limitations

As stated above, many different studies, including randomized controlled ones have been completed in the last years providing new hope for patients with PBC. However, most of them suffer from significant limitations. Patients included in these studies are UDCA-experienced patients with significantly high, more than 1.67 x upper limit of normal (ULN), levels of ALP, with few of them being cirrhotic. With the treatment target changing from lowering to normalizing ALP many patients with mildly high serum ALP levels (considered nowadays as non-responders) were excluded from these studies. Likewise, patients with decompensated cirrhosis were not included in these cohorts. Moreover, the duration of these studies is quite short for a slowly progressing disease, like PBC. Lastly, no studies have incorporated liver biopsy in their methodology; gaining insight in liver inflammation during treatment would add to understanding PBC pathogenesis.

Future research should aim to conduct larger, multicenter studies with standardized endpoints and longer follow-up, incorporating patient-centered outcomes such as quality of life, pruritus severity, and fibrosis progression. Moreover, the use of liver biopsy in selected cohorts could improve our knowledge of disease pathogenesis, while combination or sequential treatment strategies targeting different pathogenic pathways may also offer improved efficacy and warrant systematic investigation.

## 5 Conclusion

Advancements in understanding the complex pathogenesis of primary biliary cholangitis (PBC) have been instrumental in shifting treatment paradigms from symptom management to targeted therapies aimed at slowing disease progression. Insights into immune dysregulation, cholestatic injury, and bile acid homeostasis have facilitated the development of agents targeting specific pathways, including FXR agonists, PPAR agonists, and immunomodulators.

While ursodeoxycholic acid (UDCA) remains the cornerstone of first-line therapy,Elafibranor and Seladelpar have expanded treatment options for patients with an inadequate response to UDCA.

Ongoing research highlights the potential of combination therapies and emerging agents that address immune response, inflammation, and fibrosis, offering hope for improved long-term outcomes in PBC management. Among the newer agents, drugs targeting the FXR and FGF-19 pathways, alongside PPAR agonists, seem to confer the best results in phase 2 trials, even though FXR-treatment pruritus is still a concern. On the other hand, anti-inflammatory, monoclonal antibodies have shown only modest results, questioning the importance of significant inflammation in the natural course of the disease.

Overall, these results highlight the importance of a multifaceted approach to PBC, underscoring the need for continued exploration of its pathogenesis to identify novel therapeutic targets and improve patient quality of life.
